# Towards an integrative approach of healthcare: implementing positive health in three cases in the Netherlands

**DOI:** 10.1186/s12913-024-11247-x

**Published:** 2024-08-02

**Authors:** Ankie de Bekker, Maarten Beijer, Lidwien Lemmens

**Affiliations:** National Institute for Public Health and the Environment (RIVM), Centre for Nutrition, Prevention and Health Services, Bilthoven, The Netherlands

**Keywords:** Positive health, Intersectoral collaboration, Integrated care, Healthcare, Welfare

## Abstract

**Background:**

The healthcare system is under tremendous pressure. One possible solution towards relieving some of this pressure is to use Positive Health, which takes ‘health’ as a starting point, rather than ‘illness’. Positive Health provides opportunities for stimulating integrated care.

**Methods:**

Three cases in the Netherlands are studied in this paper. Their way of working with Positive Health is investigated through semi-structured and narrative interviews, using realist-evaluation and thematic analyses.

**Results:**

Seven ‘working elements’ are identified that enhance the chances of successfully implementing Positive Health in practice (part 1). The interviews show that healthcare professionals have noticed that people adopt a healthier lifestyle and gain a greater degree of control over their own health. This boosts job satisfaction for healthcare professionals too. The organisations and professionals involved are enthusiastic about working with Positive Health, but still experience barriers (part 2).

**Conclusions:**

The results of this study imply that implementing Positive Health in practice can facilitate collaboration between organisations and professionals from different disciplines, such as healthcare, welfare, and municipal health services. Operating from the perspective of a shared goal, professionals from different disciplines will find it easier to jointly organise activities to foster citizens’ health. Additionally, more attention is paid to non-medical problems affecting people’s well-being, such as loneliness or financial problems.

**Supplementary Information:**

The online version contains supplementary material available at 10.1186/s12913-024-11247-x.

## Background

The healthcare system is under tremendous pressure. High healthcare expenditure due to population ageing and an increase in chronic diseases are a challenge on a global scale [1]. Moreover, shortages on the labour market are a growing threat to the accessibility of care [2–4]. This raises the question whether our healthcare system and way of working are sustainable. In 1948, the World Health Organization (WHO) defined health as ‘a state of complete physical, mental, and social well-being and not merely the absence of disease or infirmity’. This definition is increasingly criticised [5].

More recently, broader concepts of health have been adopted [6]. For example, the widely used concept of Positive Health, which was conceptualized by Machteld Huber et al. in 2011 [7]. Positive Health entails taking a broad perspective on health; no longer considering health a static condition, but rather as the dynamic ability to adapt and manage one’s own well-being [8]. This movement has led to a bigger emphasis on people’s abilities rather than their limitations [6]. Positive Health is one of the many person-centred concepts that focus on promoting health, behaviour and functioning [9], similar to the International Classification of Functioning, Disability and Health (ICF) [10], Whole Health approach in the United States [11], Healthy Ageing by the WHO [12] and Meikirch Model of Health [13].

Positive Health provides insight into the different domains influencing health, such as: mental health, participation, daily functioning, and quality of life [14]. To create a healthcare system and an environment which stimulates healthy behaviors, a shift in focus is needed from disease management towards health promotion [15]. In the Netherlands, the well-known and widely used concept of Positive Health has, according to research, the potential to drive change both at the system level and healthcare practices by changing the focus from sickness and care to health and prevention [6]. Positive Health is a new concept that induces a transition in the perspective on health [16].

A broad perspective on health can facilitate collaboration between different organisations and disciplines by providing a shared goal and shared language [17]. In western countries, there is a focus on diminishing the gap between healthcare systems and social care services [18, 19]. For policymakers, Positive Health could be valuable as it can potentially bridge the gap between sectors and thereby demedicalise societal problems [14]. In addition, communication between different sectors can become easier, because a shared broad perspective on health also means more shared terminology. A common language is especially beneficial for intersectoral collaboration [20, 21].

In the Netherlands, different types of organisations have embraced Positive Health as a vision in their organisation, but at the same time they are struggling with how to adopt and operationalise it at different levels of the health- and social care system [17]. This impedes implementation [22]. Furthermore, there is a lack of research on citizens’ experiences [23] and on how Positive Health supports health promotion in practice [15]. Thus, there is an urgent need to exchange experiences and lessons learned between organisations and regions.

This study describes how three cases in the Netherlands adopted Positive Health into their way of working. These cases are located in three regions: The province of Flevoland, the municipality of Texel and the Leidsche Rijn-Vleuten de Meern district in Utrecht. This study’s aim is two-fold: (1) To identify working elements for adopting and implementing Positive Health in practice. (2) Explore the experiences of working with (or getting exposed to) Positive Health among citizens, professionals, and policymakers.

## Methods

### Design

The adoption of Positive Health was evaluated according to the principles of a case study design. In accordance with this design, data were collected from three cases in different ways (including interviews, learning sessions, and document analysis) and from different perspectives (including citizens, professionals, policymakers, and administrators) [24]. This evaluation is written in accordance with the Consolidated criteria for reporting qualitative studies (COREQ) [54], with a checklist available in supplementary material C.

### Positive health

Positive Health is a vision of health, where health is described as ‘the ability to adapt and self-manage’ [7]. Positive Health aims to enhance citizens’ strength and self-reliance and consists of six dimensions and 32 underlying indicators of health, which together encompasses a holistic view of health. The dimensions are: Bodily functions, mental functions & perception, spiritual/existential dimension, quality of life, social & societal participation, and daily functioning [14]. The dimensions are visualized by means of ‘the spider web diagram’. This diagram can also be used as a dialogue tool to address individuals’ health from a broader perspective, and discuss their needs, values, and preferences. A conversation based on the dialogue tool is also known as ‘the alternative dialogue’ (het andere gesprek) in The Netherlands [25]. Positive Health can also be seen as a (social) movement towards more intersectoral collaboration and prevention. For this study, cases that used similar visions or concepts to this movement, like the 4-Domains model (4D-model) [20, 21, 26], the BigMove (from ZZ to GG) [27, 28] and Right Care In the Right Place (de Juiste Zorg Op de Juiste Plek) [29, 30], were also examined. For readability, these are all described as Positive Health in this article.

### Study population

Three cases in different regions in The Netherlands were selected. All three cases are engaged in implementing Positive Health as a vision. Selection criteria for the cases included: adopting Positive Health as a vision at different levels (system-, organisational-, professional- and patient-level); focus on at least one of the following target groups: older people, people with chronic conditions, people with low socio-economic status (SES) or youth; involvement of parties from different sectors (for example: healthcare, welfare, living environment, sports); intention to self-evaluate; motivation to learn; willingness to share evaluation results and exchange learning experiences; and a contact person (e.g. project leader) with mandate to cooperate with the researchers. The selected cases are: Well in Flevoland (WEL in Flevoland), Healthy Texel 2030 (Gezond Texel 2030), and Healthy Neighborhood Alliance Leidsche Rijn Vleuten-de Meern (Gezonde Wijkalliantie Leidsche Rijn Vleuten-de Meern). More information is described in Table 1 below.

**Table 1:** Overview of three cases adopting positive health

Case	Well in Flevoland	Healthy Texel 2030	Healthy Neighborhood Alliance Leidsche Rijn Vleuten-de Meern
Region	A) Province/ county (mixed rural/ urban area)	B) Municipality/ island (rural area)	C) Neighborhood (urban area)
Population	416.000	13.000	93.000
Challenges	Aging population, social inequality, unhealthy lifestyle, rising demand of care	Aging population, rising demand of care, shortages in: labour market, volunteers and informal carers	Rising amount of new (young) citizens, social loneliness
Interventions	Social prescribing, training in Positive Health for professionals, healthy lifestyle (sport, nutrition and healthy ageing), CLI^1^	Icelandic prevention model, neighborhood de Tuunen, Social prescribing, women support group, training in Positive Health for professionals, health market at primary school	Training in Positive Health for professionals and citizens, lifestyle consultations, community building (‘Wij(k) bouwen’), CLI^1^
Parties/ collaboration	Municipal health service, nature education, art centre, sport, GPs^2^, health centres and the patient federation	Welfare, pharmacy, physiotherapists, district nursing care, sport, housing corporation, state forestry, care farm, citizens association	Healthcare centre, neighborhood platform for citizens, community centre, mental healthcare, sport, district nursing care, youth care, welfare

^1^Combined Lifestyle Intervention

^2^General Practitioners

In all three cases, the sense of urgency to search for a more sustainable healthcare system is high. The demand for care in these cases is growing strongly due to different challenges (Table 1) and cannot be met in the healthcare sector alone. Cases choose to implement interventions that are in line with Positive Health. Including interventions that increase neighborhood cohesion and lifestyle interventions. One of these interventions concerns ‘social prescribing’, this approach involves referring individuals to non-medical services and support in the community [31]. But its application is highly heterogeneous and the term is used to refer to a variety of activities [32]. As shown in Table 1, two cases have interventions similar to social prescribing, where one focuses on well-being (Welzijn op Recept) and the other on exercise (Bewegen op Recept). Another intervention is the Combined Lifestyle Intervention (CLI) (Gecombineerde Leefstijlinterventie), which focuses on reducing energy intake, increasing physical activity, and behavioral change, with the aim of reducing obesity [33, 34]. It is unknown which specific CLI programs were implemented in the cases.

The evaluation consisted of two parts: (1) the change process and (2) experiences. The first part aimed to identify working elements for adopting and implementing Positive Health in practice. The second part aimed to explore the experiences of working with (or getting exposed to) Positive Health among citizens, professionals, policymakers, and directors.

### Part 1: adopting positive health in practice: the change process

#### Interviews

To identify working elements for adopting and implementing Positive Health in practice, interviews were held at the beginning of 2020 with a number of representatives of the various participating parties in the cases (n = 11). These interviews were then repeated at the end of 2021 (n = 7). Table 2 shows a list of the interviewees. Table 3 provides an overview of the main topics. Transcripts were made of the interviews and analysed through ‘realist evaluation’ (RE) [35]. An important aspect of RE is the idea that interventions work differently in different contexts. This means that applying Positive Health can be successful in some contexts and not in others [36]. The coding and analysis of the transcripts was done by three researchers using MAXQDA (version 2022). The analysis of the first round of interviews according to the RE method resulted in context-mechanism-outcome configurations (CMOs) [35]. After de second round of interviews, the CMOs were completed and refined. The CMOs from all three cases were listed and the researchers discussed overlapping CMOs. Subsequently, CMOs were thematically clustered based on keywords in those mechanisms. Effective mechanisms were used to drawn up so-called ‘working elements’ for adopting Positive Health. Researchers also checked whether each working element was based on mechanisms from more than one case to ensure the elements were transferable across different contexts. The working elements were presented to the cases in the first learning session for verification and fine-tuning.

AB, MB and LL jointly conducted all interviews for parts 1 and 2. LL is a female senior qualitative researcher with a PhD. MB is a male researcher with experience in qualitative research (MSc). And AB is a female researcher with experience in qualitative research (MSc). None of the researchers had a previous working relationship or prior research collaborations with any of the study participants. Prior to the interview, participants were contacted to arrange a convenient time for the interviews. The duration of the interviews varied between 45 and 90 min. Field notes were taken during the interviews.

**Table 2:** List of interviewees part 1 in 2020 and 2021

Case	Organisation	interview 2020	interview 2021
A			
	Municipal health service,	yes	yes
	Center for social development	yes	no
	Province	yes	no
B			
	Municipality	yes	yes
	Municipality	yes	no
	Citizens association	yes	yes
	District nursing care	yes	yes
C			
	Neighborhood platform for citizens	yes	no
	Healthcare centre	yes	yes
	Community centre	yes	yes
	Municipality	yes	yes

**Table 3:** Interview topics part 1: adopting positive health in practice: the change process

General topics
• General experiences with Positive Health (or a similar concept) within the organization/ initiative/ case
• Recent development in applying Positive Health
• Current situation: how to apply Positive Health and what are the experiences
• Involved parties: experiences with collaboration, citizen involvement
• Evaluation: why, how, results, outcome- or process measures, indicators
• Lessons learned with implementation and future learning needs
• Future plans: developing positive health interventions or activities, long-term ambition

Each interview topic was explored on CMO configurations, by focusing on why choices were made (context), what the expected effects are or have been (outcome) and what was done to achieve them (mechanism)

### Learning sessions

Learning needs, challenges, and solutions were addressed in multiple learning sessions. The aim was to jointly draw lessons from the experiences gained in the cases. Representatives of the three cases were present at each session. Participants from the session were partly also the interviewees from Part 1 (Table 2), supplemented with other interested people from that region, such as a pharmacist and municipal policy officer. The learning sessions were supervised by a process facilitator. Researchers from the National Institute for Public Health and Environment (RIVM) observed and recorded the discussion in order to use it as a data source. Five online learning sessions were held over a two-year period (2020–2021). Except for the kick-off meeting, each learning session was substantively designed in consultation with the three cases.

### Document analysis

Different types of documents were collected from the cases: policy plans, program plans, minutes of regional consultations (for example from the steering committee from a local health program) and evaluations. These were used to supplement the collected data in the interviews and learning sessions.

### Part 2: adopting positive health in practice: the experiences

#### Narrative interviews

Narrative interviews were conducted to gain insight into the experiences of working with (or getting exposed to) Positive Health among citizens, professionals, policymakers, and directors. Interviews were held from the end of 2020 until halfway 2021. A total of 35 people were interviewed. Table 4 provides an list of the interviewees. Citizens included older people and people with psychological vulnerability, professionals included a.o. paramedics, district nurses, and social workers. The policymakers often came from municipalities and directors/administrators represented municipal health services and care and welfare organisations. With this diversity of interviewees, the width of experiences from different perspectives was taken into account as much as possible. In these interviews, the focus was on the changes that the interviewee experienced (or expected to experience) when applying (or getting exposed to) Positive Health, in which the interviewee’s story was leading [37].

**Table 4:** List of interviewees part 2

Case	Respondent	Number	Total
A			11
	Citizen	2	
	Professional	5	
	Policymaker	2	
	Director/administrator	2	
B			15
	Citizen	4	
	Professional	8	
	Policymaker	1	
	Director/administrator	2	
C			9
	Citizen	2	
	Professional	5	
	Policymaker	0	
	Director/administrator	2	

The participants were given a preparatory assignment to think about three changes they experienced after Positive Health was implemented. For example, changes in their own work, the organisation, the contact with patients or the view on their own health. Table 5 provides an overview of the main topics. Interviews took place face-to-face, by telephone or online. All interviews were audio recorded, transcribed verbatim and thematically analysed by two researchers.

independently of each other, again by using MAXQDA (version 2022). Thematic analysis focused on experiences of individual interviewees. Results were described based on different perspectives: citizen, professional, policymaker, and director.

**Table 5:** Interview topics part 2: adopting positive health in practice: the experiences

General topics
• What means ‘health’ to you?
• When was your first introduction to the vision of Positive Health?
• What has changed in your life or work as a result of working with Positive Health?
• The three changes experienced after Positive Health was implemented according to the respondent (preparatory assignment)

## Results

### Part 1: working elements

Based on the analyses on the three cases, seven working elements for adopting Positive Health emerged. For each element, it is described ‘how’ adoption was achieved or how it was intended to be achieved. Appendix A describes the working elements in more detail and Appendix B includes examples of underlying CMOs.

#### Commitment from all parties involved

The interviewees experienced that not all stakeholders readily commit to a broad perspective on health such as Positive Health. Sometimes there was even resistance to (applying) the concept. In order to nevertheless create support and motivation, various strategies and activities have been deployed. On organisational level, one case has deliberately chosen a name that implies a broad view of health, rather than specifically referring to Positive Health. There was also a ‘coalition of the willing’ in each case, i.e., a group of people who already embrace the concept and want to put it into practice. Subsequently, inspiration sessions and network meetings for professionals were organised in which experiences and success stories about applying Positive Health were shared. As it takes a long time to make the transition towards a broader perspective on health, patience and awareness were found to be important to sustain commitment.

#### A clear focus within the approach

Positive Health covers everything related to health, from physical health to the meaning of life and participation in society. The interviewees from each case experienced that this complicates setting specific goals and choosing themes and activities. Therefore, interviewees use regional data on population health and well-being to gain insight into the most urgent problems to determine relevant themes and activities. All three cases reflected with the collaboration partners on the goals and ambitions and these were adjusted if necessary.

#### Professionals knowing and understanding one another

Interviewees from all three cases experienced that for a good intersectoral collaboration, it is important that professionals from different organisations get to know and understand each other. Encounters between professionals were facilitated in each case. For example, by organising joint trainings in the philosophy of Positive Health. On organisational level, thoughts are given about how to facilitate cross-sectoral meetings now and in the future in a structural way. All three cases were working on low-threshold meeting places for professionals in the neighborhood (e.g. health center).

#### Work from citizens’ needs and possibilities

In all three cases, organisations try to take into account the needs and possibilities of citizens as much as possible. For this reason, they cooperated with local citizens/volunteer organisations. At the same time, interviewees experienced that citizens’ organisations do not always have sufficient manpower and organisational experience, which has sometimes limited their contribution. Another way to work from citizens’ needs and possibilities is to have *the alternative dialogue* between professionals and their patients. In all three cases, professionals are trained in this conversation method and learn to look beyond the (medical) complaints to find out what needs and possibilities their patients really have.

#### Provide a facilitating organisational structure

On organisational level, a facilitating structure can be achieved by setting up an organisational structure that supports collaborating around applying Positive Health. Stakeholders from all three cases stated that their organisational structure is set up in such a way. For example, a multi-layered structure has been chosen with a decision-making and/or management layer (e.g. steering group) and an executive layer (e.g. working group). In addition, analyses revealed the importance of continuity in program management.

#### Ensure financial resources to achieve goals

In order to achieve the intended goals, more structural funding is needed for activities based on Positive Health. All cases intend to use national or regional grants. Furthermore, in all cases, attempts were made to achieve more sustainable financing agreements with municipalities and health insurers. However, this is still work in process. Moreover, some partnerships focussed on shared savings, while others request contribution from collaboration partners.

#### Embed positive health more in society

In all three cases, efforts were made to embed a broad view on health in society, thereby attempting to make the adaptation of Positive Health more sustainable. In both primary and secondary education and vocational training, attention is paid to Positive Health. Moreover, in all three cases, efforts were made to increasingly refer citizens to regular sports and exercise programs instead of programs within the healthcare sector. Furthermore, professionals aim to make citizens more aware of their own influence on health by using a dialogue tool. From the interviewees’ perspective, it helps if the focus is more on health than disease among national and regional policymakers (health in all policies).

### Part 2: experiences from the field

The narrative interviews gave insight into changes resulting from applying Positive Health. Experiences are described from different perspectives: Directors, policymakers, professionals, and citizens.

### Directors and policymakers

The application of Positive Health in the studied cases was relatively recent. According to some interviewees, it is therefore too early to observe improvements. However, the interviewees found that working with Positive Health does bring about changes. Since there is a broader view on health than solely a medical perspective, more opportunities are created for activities and/or interventions focusing on prevention and other aspects of life.

*“Because it [Positive Health] allows you to look at multiple aspects of human health, and at the balance between various aspects. I think it helps us get a more open mind. That we are not focused on the problem and the self-evident solution.” (Director)*

Changes are also noticeable at the organisational level. Organisations spend more time getting to know other stakeholders by organising inter-professional and intersectoral meetings. Interviewees expect that this can lead to fewer referrals to secondary care. For example, a healthcare professional said that less medication is prescribed for chronic diseases and that (instead) referrals to lifestyle interventions are made more often.

Within healthcare and welfare organisations themselves, applying Positive Health has resulted in more attention to the well-being of employees. For example, mindfulness courses are offered, or employees are given a budget to make healthy choices such as buying a bicycle. According to a number of interviewees, this increases health awareness among employees, leading to healthier choices. For example, employees more often cycle to work or go for a walk during lunch, leading to lower absenteeism due to illness. Administrators hope that healthcare professionals will also incorporate this health awareness into their work, in such a manner that they can encourage patients to think more about their own health too.

### Professionals

Professionals working according to the philosophy of Positive Health say they collaborate more, both within and across sectors. For example, a GP explained that she is looking more closely at the possibilities for patients within the social and welfare sector.

*“What you often hear is: ‘we are going to ask things that do not belong to a doctor’. And I totally agree with them. But by doing that, you can ensure that the patients’ question ends up in the right place, isn’t it? Trying to find the solution. I like that very much.” (Professional)*

Furthermore, professionals working with Positive Health point out that they have a common language and way of thinking, resulting in better and faster mutual understanding. A number of professionals emphasize that it would be valuable for the patient if all (healthcare) professionals collectively work with Positive Health. This is not yet the case in the studied regions.

Some professionals also experience a reappraisal of their work, as Positive Health provides a sense of underpinning for how they already worked. Likewise, many professionals indicated that they experience more job satisfaction by working from a broad health perspective. They feel more connection with the patient and learn more about the patient.

*“She [the patient] filled in the spider web and found out that she didn’t have much time for social contacts. And it became clear that she might have had more stress than she actually experienced. And by focusing more on actually relaxing and doing social things, her blood pressure also went down.” (Professional)*

Because patients themselves are stimulated to think about different aspects of life, the underlying problems often become clear more quickly. Healthcare professionals noticed that other aspects of life, such as financial problems, have a great influence on the well-being of the patient. Professionals are now more aware of this, partly due to the newly learned conversations methods. In addition, patients seem to gain more insight into their own situation and more often come up with solutions themselves.

*“In the [consultation room of the] GP, when people are bothered by something, they often sit across the desk and say: ‘I’m bothered by this or this is annoying, solve it for me’. But I notice that if I give them the spider web [conversation tool], they also start to think more consciously on their side, like what can I do, and already think about solutions or ideas where it [the symptoms] might come from.” (Professional)*

The patient’s role in the consultation room thus changes from passive to active according to several interviewees. This also makes the conversation feel less burdensome for the healthcare professional. It is often thought that conversations can be lengthy when using the alternative dialogue. However, one healthcare professional mentions saving time after using it once in the initial consultation.

### Citizens

The interviews show that citizens, who participated in one of the activities based on Positive Health (see Table 1), largely share a positive view on the concept, which corresponds to the professionals’. They also feel that their complaints are looked at more broadly, for example, when they participate in an exercise program or talk to a social worker. In the various activities that take place in the cases, the emphasis is often on giving meaning to life and participation. For example, in one case, people with mental disorders are brought into contact with an art organisation. GPs in another case refer citizens more often to the social worker. People can thus end up in various welfare activities. Citizens indicate that these kinds of initiatives make them feel less lonely or stressed. They have a sense of belonging somewhere again. In addition, citizens in all three cases experience more control over their health.

*“Because you gain experience [at the support group] by talking mutually about it. And you process all difficulties that you have had yourself, which I think is also very important. […] You get a completely different view of the world. And on your own life. And how to deal with it when there are difficulties.” (Citizen)*

Another example comes from a Combined Lifestyle Intervention (CLI), where participants experience that fewer (behavioural) rules or orders are imposed by the lifestyle coaches compared to regular fitness or nutrition programs. Through stimulating health awareness, the program is more effective for them than a regular program.

*“So he ensures that you can continue independently. Because, well, I’ve been to a dietician many times and when you ask, she gives it. You ask for recipes, she makes and finds the recipes for you. But he [CLI trainer] says, you really have to do it yourself […] I started figuring out what I want to eat, how much I can eat, what I should leave out. […] It just motivates me to do it myself.” (Citizen)*

Awareness about healthy choices is a recurring theme during the interviews. People discover they are capable of more than they expected beforehand. They feel more empowered about their health.

### When does it not work?

There are also objections to the use of Positive Health. Some professionals found the courses about Positive Health unnecessary or experienced that the new conversation method takes considerable time. Likewise, it does not work for every persons’ complaint.

*“People were simply overloaded with the concept of Positive Health. And they thought: ‘hey, I really don’t feel like this at all…’ I don’t think Positive Health is the goal, you know. It is a way to help you in your conversation. And it is a method that you really do not always need.” (Professional)*

Even among citizens, Positive Health or alternative dialogue does not always work. A professional described that some people do not like to reflect on themselves using the spiderweb conversation tool. For example, because they find it difficult to set goals.

## Discussion

Our aim was to understand how Positive Health could be adopted in practice. And to gain insight into the changes resulting from this new way of working. We identified seven elements that enhance the chances of success when applying Positive Health: commitment from all parties involved; a clear focus within the approach; professionals knowing and understanding one another; work from citizens’ needs and possibilities; provide a facilitating organisational structure; ensure financial resources to achieve goals; embed Positive Health more in society. Furthermore, we found that a broad perspective on health can facilitate collaboration between organisations and professionals from different disciplines, such as healthcare, welfare, and municipal health services. Operating from the perspective of a shared goal, interviewees found it easier to organise activities to foster citizens’ health and well-being.

### Setting into international and evidence context

The seven working elements can contribute to the adoption of Positive Health in practice and are in line with the international literature about successful strategies for cross-sectoral collaboration and population health management [38, 39]. The findings provide policymakers, organisations, and (health care) professionals with an understanding of the working elements, which promote the adopting of Positive Health. The purpose of this information is to enable regions to adopt and implement their own effective Positive Health interventions.

Different experiences with the implementation of Positive Health emerged from the narrative interviews and show how Positive Health can contribute to a sustainable healthcare system and the quadruple aim [40]. Firstly, administrators and policymakers noticed that more attention is given to the employees’ well-being. In recent years, there has been more attention for the mental health needs of healthcare professionals, according to the literature. The high exposure to multiple stressors, burnout, and depression within healthcare professionals work is increasingly recognised [41, 42]. Positive Health can be used to emphasize employees’ well-being by giving space to the spiritual domain, quality of life and societal participation. Secondly, professionals in the narrative interviews stated that they collaborate more often with other sectors such as welfare. Today, health issues are increasingly complex and often have several underlying issues. A broad perspective on health, like Positive Health, creates consciousness about other factors in a person’s life and stimulates intersectoral collaboration. Positive Health is known for its widely supported ‘vision’ and common language [14]. Having a shared vision was found to be helpful for intersectoral collaboration [26]. Thirdly, professionals experienced that citizens adopt a healthier lifestyle and are given a greater degree of control over their own health. This boosts also job satisfaction for healthcare professionals. Previous research also shows that Positive Health motivates professionals and increases job satisfaction [43]. This is important to maintain our precious healthcare workers and attract new workers considering the current shortages. Fourthly, by participating in organised activities based on Positive Health, citizens themselves notice that they feel less lonely and stressed, and experience increased health awareness. With rising healthcare costs, people’s awareness of lifestyle and health is becoming increasingly important. Despite research into citizens’ experiences being so important, to our knowledge, the impact of Positive Health on citizens has been scarcely studied [23]. In outpatient consultations, using the dialogue tool seems a promising strategy to increase value-based healthcare for citizens [44]. But there are also concerns, for example, about whether everyone can handle self-management [45] or the requirements for substantial personal input [14]. Lately, professionals have expressed a desire for a more informed application and elaboration of Positive Health [46]. Currently, Positive Health is operationalised in varying ways, but without one allocated intervention [18]. As a result, a reliable measurement tool for applying Positive Health is still missing [47–49]. Without a measurement tool, it is impossible to measure quantitative effects. However, several studies on the development of a measurement tool are currently being initiated [50]. Ultimately, in our view, the different concepts will all contribute to a broader perspective on health.

### Strengths and limitations

Various interventions based on Positive Health were implemented in the studied cases. The interventions often entail cross-sectoral collaboration and are about integrating care. This research was conducted in 2020–2021, amid the Covid-19 outbreak. Newly designed interventions and activities did not start and meeting locations had to (temporarily) close their doors in the Netherlands. According to the interviewees, collaboration between organisations became more difficult because physical meetings were hardly possible. This has also influenced the results of this study. Because fewer interventions were implemented, fewer changes may be expected. Collecting data was also more difficult. This should be taken into account in the interpretation of the results. Other studies in this period also report fewer developments, because setting up integrated care interventions was challenging during the pandemic [51]. On the other hand, Covid-19 was also a driver for setting up collaborations [52] and increasing teamwork [53].

## Conclusion

Adopting a broad perspective on health such as Positive Health can contribute to integrated care on the patient-, professional-, organisational- and system level. Several working elements were identified. Furthermore, this study provides insight into the experiences of various stakeholders and citizens. Our study suggests that Positive Health may be promising in regard to creating a sustainable healthcare system and may lead to health benefits for both professionals and citizens. However, developing a reliable measurement tool is essential. Moreover, a large-scale study on the effects of different activities and interventions based on Positive Health should be carried out.

### Lessons learned

- Determine a clear focus when applying Positive Health in practice. Together with collaborating partners, choose which interventions contribute to your goals.
- Have a different conversation with citizens, what are their questions and needs? These often seem to lie outside the healthcare system. But also pay attention to citizens for whom this method is not suitable.
- Truly embedding into society means focusing on education and connecting with what residents are already organizing themselves.

### Electronic supplementary material

Below is the link to the electronic supplementary material.

Supplementary Material 1

Supplementary Material 2

Supplementary Material 3

## Data Availability

Data are not publicly available to preserve participant anonymity.

## Abbreviations

CLI: Combined Lifestyle Intervention
CMO: Context-mechanism-outcome
COREQ: Consolidated criteria for reporting qualitative studies
GP: General Practitioner
ICF: International Classification of Functioning, Disability and Health
RE: Realist Evaluation
RIVM: National Institute for Public Health and the Environment
SES: Socio- economic status
WHO: World Health Organization
