# Supplementary material for: Towards an integrative approach of healthcare: implementing positive health in three cases in the Netherlands
Source: BMC Health Serv Res. 2024 Aug 2;24:882. doi: 10.1186/s12913-024-11247-x (PMC11295315; doi:10.1186/s12913-024-11247-x)
Supplement: Supplementary file 1 — Supplementary Material 1 [file 12913_2024_11247_MOESM1_ESM.docx]

**Appendix A. Working elements for adopting and implementing Positive Health in practice.**

| **Working elements** | **Mechanism (How to achieve adoption?)** |  |
| --- | --- | --- |
|  |  |  |
| Commitment from all parties involved | Implement proven effective interventions |  |
|  | Involve important organizations in the region (municipality healthcare services e.g.) |  |
|  | Involve strategic partners/persons and create administrative support |  |
|  | Chosen an appealing name; one that implies a broad view of health |  |
|  | Start where the energy is (‘coalition of the willing’) |  |
|  | Do not limit yourself/your organization to just one concept |  |
|  | Join existing initiatives of partners |  |
|  | Let specific partners/organizations join with specific themes |  |
|  | Increase visibility of activities and interventions |  |
|  | Share and exchange stories about what works |  |
|  | Organize inspiration sessions and network meetings for professionals |  |
|  | Use social media and online meetings during a pandemic |  |
|  | Focus on interventions that are easily accessible for professionals |  |
|  | Have patience, this transition takes a long time |  |
| A clear focus within the approach | Create a shared vision |  |
|  | Choose themes and activities based on regional data |  |
|  | Reflect on goals and ambitions with the collaboration partners |  |
|  | Recognize fragmentation and loss of focus |  |
|  | Make choices in grant applications, projects and interventions |  |
|  | Leave the choice of using a dialogue tool with the professional |  |
|  | Think about whether and how to use a broad health concept for each project |  |
|  | Focus on citizens' needs and capabilities |  |
| Professionals knowing and understanding one another | Use online meeting tools to stay connected as partners |  |
|  | Let professionals from different organizations collaborate in a shared working environment |  |
|  | Let professionals from different organizations be part of (cross-sectoral) activities and projects |  |
|  | Create an informal atmosphere/setting during meetings |  |
|  | Use Positive Health as a common language for professionals |  |
| Work from citizens’ needs and possibilities | Cooperate with local citizen and volunteer organizations |  |
|  | Organize an awareness campaign for citizens: about resilience and influence on their own health |  |
|  | Offer citizens a platform for initiatives |  |
|  | Train professionals and volunteers in a broad health concept |  |
|  | Take into account the limited organizing capacity of citizens/volunteers |  |
|  | Let citizens use their talents when it comes to volunteering |  |
|  | Make sure you connect with what is possible for the citizens |  |
|  | Introduce citizens to a broad health concept in a low-threshold manner |  |
|  | Improve conversations between professionals and clients through the alternative dialogue |  |
|  |  |  |
|  |  |  |
| **Working elements** | **How to achieve adoption?** |  |
|  |  |  |
| Provide a facilitating organizational structure | Set up an organizational structure that supports collaborating regarding applying broad health concepts |  |
|  | Use a multi-layered structure with a decision-making and/or management layer (e.g. steering group) and an executive layer (e.g. working group) |  |
|  | Choose a matching legal form, depending on your goals. Or change the legal form when necessary |  |
|  | Use alternative ways to hold meetings, and less is more. |  |
|  | Ensure the right type of project leadership and continuity in program management |  |
|  | Use broad health concepts in personnel policy/ performance review |  |
|  | Use a communication platform for professionals from different disciplines and organizations |  |
| Ensure financial resources to achieve goals | Use money flows from national or regional grants |  |
|  | Make financing agreements with municipalities and health insurers about their contribution |  |
|  | Invest saved healthcare costs (shared savings) |  |
|  | Integrate broad health concepts in municipal health policy |  |
|  | Request contribution from cooperation partners (paid or unpaid) |  |
|  | Make sure the organization and infrastructure investments are covered for intersectoral collaboration |  |
| Embed broad health concepts more in society | Focus more on health than disease among national and regional policy (health in all policies) |  |
|  | Ensure embedding in vocational training, but also in primary and secondary education |  |
|  | Connect with existing citizens' initiatives or activities |  |
|  | Make citizens more aware of their own influence on health |  |
|  | Increase referring citizens to regular sports and exercise programs |  |
|  | Encourage citizens to take responsibility for their own health |  |
