# Supplementary material for: Towards an integrative approach of healthcare: implementing positive health in three cases in the Netherlands
Source: BMC Health Serv Res. 2024 Aug 2;24:882. doi: 10.1186/s12913-024-11247-x (PMC11295315; doi:10.1186/s12913-024-11247-x)
Supplement: Supplementary file 2 — Supplementary Material 2 [file 12913_2024_11247_MOESM2_ESM.docx]

**Appendix B. Example of CMOs**

|  | **Commitment from all parties involved** | | |
| --- | --- | --- | --- |
|  | Context | Mechanism | Outcome |
| 1 | Region B chose to work with interventions that have proven effective through research, like social prescribing (WOR). | No administration of indicators in the GP practice is necessary for research purposes as the intervention is already proven to be effective. Therefore, GPs do not have to do extra administration. The projectleader focusses on keeping GPs connected and enthusiastic. | GPs are motivated to participate in this intervention. GPs are not necessarily spending more time in consultations or administration. |
| 2 | Region A wanted to adopt PH into the vision of their coalition, but experienced resistance from partners when PH was used in their coalition name. Even though the partners agree on the philosophy of PH ('enhancing well-being') some partners do not like the language and terminology used within PH. | Instead of sticking to one specific concept and its name, partners together chose a more appealing name; one that implies a broad view of health | Name of the coalition includes the term 'wellbeing', in which all partners recognize themselves. |
| 3 | Citizens from region B are concerned about upcoming health challenges (an aging population, medical care is scarce, labor market). As a citizens' initiative they started looking for ways to solve this challenge. Through contact with the health insurer (innovation team) the concept of PH got introduced | During a lecture about PH many citizens and organisations became enthusiastic and motivated, they formed a 'coalition of the willing' on the spot. | The coalition was the driving force/engine behind a bigger cooperation/change. It enhanced intrinsic motivation in the people involved. |
|  | **A clear focus within the approach** | | |
|  | Context | Mechanism | Outcome |
| 4 | In Region C the Neighborhood Alliance wants to expand by including new partners but at the same time they want to get started quickly. Discussions are held with various partners to explore how to shape cooperation. The Alliance wonders if there is enough support for expansion. | Guided by an external consultancy company on the alliances' approach, discussion were held about the goals and vision. | Support for the program has grown stronger through discussions. They concluded that they need to prevent fragmentation and loss of focus. Therefore, the working group has been made smaller instead of larger. Each year they want to focus on one major theme. |
| 5 | In Region C they conduct the 'alternative dialogue' with patients and citizens. However, different broad health concepts are used in the region, and therefore also different interview instruments. For example, the social sector works with the four domains (4D) model and with PH. | The region has decided that the professionals are able to choose which dialogue tool they prefer. Professionals are trained in both. As both concepts strive for the same goal (‘broad view’) the region is fine by using both instruments. | Some partners had an aversion to PH specifically, but agreed on its perspective ('looking broadly at health'); by including another broad health concept, everyone could still participate. |
|  | **Professionals knowing and understanding one another** | | |
|  | Context | Mechanism | Outcome |
| 6 | Many different disciplines are connected to the 'health center' in Region B increasing contact with each other through its consultation structure. In these consultations, they discuss domains of PH and all kinds of concepts: wellbeing, resilience, integrated thinking, etc. | All professionals become more familiar with the terminology of PH. | A common language is being developed. Different disciplines have started to use PH terminology and now understand each other faster. |
|  | **Work from citizens’ needs and possibilities** | | |
|  | Context | Mechanism | Outcome |
| 7 | The IDKG team (citizen organisation) in Region C includes 30 volunteers. All volunteers completed a workshop and basic module about PH. Anyone from the neighborhood may join the team. People are deployed based on their skills and talent. | Some citizens can devote more time to the IDKG team than others. But even when they can spend little time on it, they meet new people as a result. Attending the Positive Health module has a positive impact on the team and the work they do together. | Citizens are regaining confidence in their own abilities. Citizens' social network enlarges. The volunteers promote PH to their other contacts, and apply it in their own lives. The volunteers think in possibilities instead of problems. |
| 8 | The GP in Region C started using the 'alternative dialogue' method (based on PH) with all its patients during consultations. | The 'alternative dialogue' with the patient, focusses more on coaching and restrains the urge to treat immediately. Citizens' own problem-solving abilities are addressed more. | Conversations between professionals and patients are improved. According to the GP, patients feel heard. Further, the GP experiences greater job satisfaction by using the 'alternative dialogue'. |
|  | **Provide a facilitating organisational structure** | | |
|  | Context | Mechanism | Outcome |
| 9 | The project team in Region A included professionals from different levels, (healthcare) professionals, strategist, administrators. Project team members found it difficult to take decisions; strategic and implementation choices were mixed up. | Project team was split into smaller groups, using a multi-layered structure with a decision-making and/or management layer (e.g. steering group) and an executive layer (e.g. working group) | Decision-making process is improved. The project team reaches decisions faster and better. |
| 10 | Many professionals in Region B already embraced the PH philosophy and integrated it in their own way of working. Organisations, that otherwise would not work together, were brought into contact with each other by creating a consultation structure and 'health center'. The projectleader tried to shape this structurally. | Funding of the coalition stopped, forcing the projectleader to quit. There was also a change of director at the welfare organisation, who appeared to be less enthusiastic about the PH concept. In addition, the local councillor changed. | It is uncertain whether the collaborations will continue without a projectleader and structural budget. The steering committee needs to reconsider program management. They question whether an external projectleader is best. |
|  | **Ensure financial resources to achieve goals** | | |
|  | Context | Mechanism | Outcome |
| 11 | In Region B attempts are made to implement the PH philosophy through action plans. The coalition tries to cooperate with the health insurer to make financing agreements about their contribution. | The coalition experiences difficulties with entering into discussion with the health insurer. The health insurer appears to have other priorities. They went on for a long time trying to cooperate with the health insurer. | Coalition had anticipations for a long time, but the health insurer ultimately did not want to contribute to structural funding. The implementation of the PH action plans is again put on hold. |
| 12 | The healthcare system in the Netherlands is primarily focused on treating diseases and care, and less on health promotion and prevention. Nationally, there are only a few funds that would finance the PH movement. | In Region B there are local funds that want to contribute, and the municipality pays several interventions, but there is no extra budget. Bottom-up energy resulted in collaboration between citizen cooperation, welfare and healthcare. Further, PH is integrated in municipal health policy. | Projectleader experiences challenge around accelerating, broadening and strengthening the PH movement. Policy officer at the municipality tries to develop PH further within and outside the organisation. |
|  | **Embed broad health concepts more in society** | | |
|  | Context | Mechanism | Outcome |
| 13 | The coalition in Region A links up with existing PH initiatives by local partners. E.g. with a nature organisation, local prevention model and art project in care homes. These initiatives fit well with the PH philosophy, focus on prevention, and are therefore motivated to join the PH network. | The partners in the network like to be visible. Through the network they get inspired by others. The coalition created a digital platform for communication to be used by the partners. | The coalition connects partners from different sectors to PH and prevention. But in practice, this proves to be challenging. In the end, partners still make little use of each other’s initiatives. |
| 14 | Exercise on Prescription' and 'Wellbeing on Prescription' (social prescribing variants) are interventions deployed in the context of PH in Region B. Referrers are physiotherapy practices, other paramedics or GPs. They refer residents or patients with e.g. mild psychosocial complaints to a sports- or exercise coach. | Sports- or exercise coaches try to guide people to a regular sports and exercise programs in the neighborhood (e.g. sports club). | Through this type of intervention collaboration between professionals gets off to a good start. Taking part in such interventions could improve participants' quality of life, and potentially relieve the GP from some of its work. |
|  | *PH: Positive Health |  |  |
